# Using Next-Generation Sequencing to Contrast the Diet and Explore Pest-Reduction Services of Sympatric Bird Species in Macadamia Orchards in Australia

**DOI:** 10.1371/journal.pone.0150159

**Published:** 2016-03-01

**Authors:** Eduardo Crisol-Martínez, Laura T. Moreno-Moyano, Kevin R. Wormington, Philip H. Brown, Dragana Stanley

**Affiliations:** 1 School of Medical and Applied Sciences, Central Queensland University, Rockhampton, Queensland, Australia; 2 School of Biosciences, University of Melbourne, Parkville, Victoria, Australia; 3 Institute for Future Farming Systems, Central Queensland University, Rockhampton, Queensland, Australia; Institute of Zoology, CHINA

## Abstract

Worldwide, avian communities inhabiting agro-ecosystems are threatened as a consequence of agricultural intensification. Unravelling their ecological role is essential to focus conservation efforts. Dietary analysis can elucidate bird-insect interactions and expose avian pest-reduction services, thus supporting avian conservation. In this study, we used next-generation sequencing to analyse the dietary arthropod contents of 11 sympatric bird species foraging in macadamia orchards in eastern Australia. Across all species and based on arthropod DNA sequence similarities ≥98% with records in the Barcode of Life Database, 257 operational taxonomy units were assigned to 8 orders, 40 families, 90 genera and 89 species. These taxa included 15 insect pests, 5 of which were macadamia pests. Among the latter group, *Nezara viridula* (Pentatomidae; green vegetable bug), considered a major pest, was present in 23% of all faecal samples collected. Results also showed that resource partitioning in this system is low, as most bird species shared large proportion of their diets by feeding primarily on lepidopteran, dipteran and arachnids. Dietary composition differed between some species, most likely because of differences in foraging behaviour. Overall, this study reached a level of taxonomic resolution never achieved before in the studied species, thus contributing to a significant improvement in the avian ecological knowledge. Our results showed that bird communities prey upon economically important pests in macadamia orchards. This study set a precedent by exploring avian pest-reduction services using next-generation sequencing, which could contribute to the conservation of avian communities and their natural habitats in agricultural systems.

## Introduction

Birds are one of the most studied groups of animals on the planet because of their importance as bioindicators, providers of ecosystem services and high visibility [1, 2]. Native bird species that inhabit agricultural landscapes across the world strongly depend on native vegetation [3]. However, these landscapes are generally formed through clearing and fragmentation of native vegetation, threatening resident avian communities through reduced availability of habitat and increased genetic isolation [4]. Furthermore, bird insectivory in these agro-ecosystems is potentially disrupted by management practices including insecticide applications. Thus, while some avian species may thrive in agricultural landscapes, many other species are threatened by the global expansion of agricultural production.

Many studies across several crop systems have highlighted the positive contribution of birds to agriculture by reducing insect pest numbers [2, 5]. Although several bird species are generalist predators, their pest-reduction services can in some instances indirectly increase crop yields (e.g. [6–8]). A call was recently made to estimate the economic value of these free services [9], reinforcing the need to explore and exploit them. Diet analysis offers a first approach to assessing avian pest-reduction services in agriculture by understanding how the feeding behaviour of bird communities can impact the arthropod assemblages [10].

Traditionally, dietary studies have been conducted using techniques such as the observation of arthropod fragments in faecal samples (e.g. [11, 12]). Other traditional techniques, related to stomach content analysis, are invasive, and in some instances require killing of the animal. Furthermore, the digestion process decomposes arthropods making taxonomic identification using these techniques difficult. Where identification is possible, it rarely goes beyond the family level [13]. In contrast to the traditional methods, the development of non-invasive molecular techniques has allowed the detection of DNA remains in faecal samples, allowing precise detection and higher taxonomic resolution of both hard- and soft-bodied insects within bird faecal samples [14].

The development of next-generation sequencing (NGS) has allowed the simultaneous sequencing of millions of amplified DNA templates [10], representing a powerful yet affordable molecular technique for dietary analyses. A number of studies have investigated the diet of bats using NGS sequencing (e.g. [15, 16]). These studies effectively used NGS in combination with arthropod-specific primers [17] targeting a 157bp fragment of the mitochondrial cytochrome oxidase I (COI) region for studying the diet of insectivorous bats. Thus, this technique could be applicable to other generalists, including birds [18]. A recent review has stressed that the slow adoption (and adaptation) of these molecular methods by ornithologists [19], is leading to a lack of published studies using NGS methods in avian research. Only a few studies have used these methods to explore the diet of birds (e.g. [20]), but to our knowledge, they have never been used to study avian pest-reduction services in agriculture.

In Australia, the value that insectivorous birds may have in agriculture has not been extensively researched [21]. Most of the dietary studies in birds were conducted using traditional techniques (e.g. [22, 23]). Eastern Australia, one of the regions most affected by agricultural deforestation [24], is the world’s most productive area of macadamia, a high-value nut tree. Covering an approximate area of 19,000 ha, macadamia is a young industry that is expanding due to an increasing market demand [25]. Within this area, Bundaberg is the fastest growing macadamia region in the country [25]. Macadamia production areas in this region are composed of matrixes of orchards and other crops interspersed with forest patches, most of which are interconnected by riparian vegetation corridors. Previous studies have documented the tremendous importance of riparian habitats to avian biodiversity in Australia [26, 27]. Bird communities in these habitats potentially deliver pest-reduction services in macadamia, but their role is as yet undefined. Hemipteran pests such as *Amblypelta lutescens lutescens* (Coreidae; banana spotting-bug) and *Nezara viridula* (Pentatomidae; green vegetable bug) are considered major macadamia pests [28]. The most effective control method has been the application of broad-spectrum insecticides [29]. Alternative strategies exploiting natural ecosystem services could be used to maintain low pest levels while reducing the use of insecticides [30]. Thus, assessing avian pest-reduction services could promote orchard and landscape management practices that help reduce pest levels and support the conservation of bird communities in macadamia production areas.

In this study, we used NGS to explore pest-reduction services of birds foraging in macadamia orchards adjacent to riparian habitats in Bundaberg, Australia. Our aims were to: 1) elucidate whether the NGS techniques and protocols, using the Illumina Miseq platform, can be used to study the diet of birds, 2) describe whether bird species in macadamia orchards are feeding on insect pests, and 3) understand resource utilization in the avian community through within- and between-species dietary comparisons.

## Materials and Methods

### Study area and site

We carried out this study in the Bundaberg region in eastern Australia. Sample collection was conducted in a macadamia orchard (Lat. 25.0957° S, Lon. 152.3345° E), located approximately 25 km south of Bundaberg. This orchard was adjacent to a strip of riparian fringing forest, a situation common to most orchards in the region. Riparian habitats in this region are generally composed of a number of tree species, including *Eucalyptus tereticornis* (forest red gum), a number of *Melaleuca* (tea tree) species, often including *M*. *viminalis* (weeping bottlebrush) and *M*. *bracteata* (black tea tree), *Casuarina cunninghamiana* (creek oak), and *Waterhousea floribunda* (weeping myrtle). The region has a characteristic sub-tropical climate, with hot summers and mild winters, and annual average maximum temperatures of 26.7°C (37 years; 1959–2015) [31].

### Bird trapping and faecal sample collection

Birds were captured at the study site during 4 weeks in November of 2014. The study was timed to coincide with period when the population of major macadamia pests is highest, corresponding to the early nut development stage in the crop. Six mist-nets (approximately 3 × 9 m) were set each day and attended continuously from sunrise till mid-day and from late afternoon till sunset. To maximize the capture probability, mist-nets were evenly distributed throughout the orchard, parallel to the macadamia rows, and their positions were changed every week. Each trapped individual was carefully removed from the mist-net, identified, and then placed individually in a sterile cloth bag for 30 minutes. After this time, the bird was released, and faecal material in the cloth bag collected using a new, sterilized plastic spoon for each faecal sample. Cloth bags were used only once before soaking in hypochlorite solution for 24 hours, washing thoroughly and sterilising for the next use. Each sample was transferred to a 5 ml plastic tube filled with 95% ethanol and immediately placed in a refrigerated container in order to preserve DNA integrity [32]. At the end of each day, all collected samples were stored at -20°C. Samples were stored under these conditions for one month until DNA was extracted. To minimize the risk of contamination during field data collection, all materials used were washed each day in a solution of 5% hypochlorite.

### Animal ethics statement

Animal ethics approvals were obtained from the Animal Ethics Committee of Central Queensland University (project no. A14/05-310) and the Department of Environment and Heritage Protection of the Government of Queensland (permit no. WISP14680814).

### Insect pest surveys

Parallel to the bird trapping, we used two sampling methods to monitor the presence and collect individuals from two of the major macadamia insect pests. Pheromone traps were used to capture *A*. *lutescens lutescens* and sweep-net samplings were performed over the macadamia tree canopies to capture *N*. *viridula*.

### Faecal sample preparation, DNA extraction and quantification

Prior to weighing the faecal samples, seeds, fruits and feathers were removed. This cleaning process was performed on one sample at a time to avoid DNA degradation due to an increase in temperature [33]. Wet weight of each sample was obtained after it was centrifuged for 30 seconds at 5,000xg and the supernatant ethanol removed. To ensure the homogenization of samples and maximize DNA recovery, 1 tungsten carbide bead (3 mm) was placed into each 1.5 ml Eppendorf tube containing a sample and these were frozen by submersion in liquid nitrogen for 10 minutes. Frozen tubes were immediately placed in a high-energy, high-throughput cell disrupter (Mini-beadbeater-96, Biospec) at 2,100 oscillations/min for 1 minute.

DNA was extracted from faecal samples using QIAamp DNA Stool Mini Kit (Qiagen, #51504) following the manufacturer’s instructions, with the modifications proposed by Zeale *et al*. [17]. Additionally, insect DNA was extracted from two major macadamia insect pests, *A*. *lutescens lutescens* and *N*. *viridula*, using DNeasy Blood and Tissue Kit (Qiagen, #69581), using legs from individuals of each species trapped during the insect pest surveys. Negative controls were included in both DNA extractions to ensure no cross-contamination occurred. Afterwards, DNA was quantified using a full-spectrum spectrophotometer (NanoDrop 2000, Thermo Scientific) and then stored at -20°C until PCR analyses.

### DNA amplification of inspect pest and faecal samples

Arthropod-specific primers ZBJ-ArtF1c (Forward: AGATATTGGAACWTTATATTTTATTTTTGG) and ZBJ-ArtR2c (Reverse: WACTAATCAATTWCCAAATCCTCC) [17] were used to amplify a 211 bp section (54 nt primers and 157 insert) of the COI mitochondrial DNA barcoding region. Prior to the experimental use of these primers, we first evaluated the efficiency of the primers in *A*. *lutescens lutescens* and *N*. *viridula*. Insect pest PCRs were run in 25 μl reactions. Each PCR reaction contained 2 ng/μl of DNA template, 12.5 μ KAPA Hotstart polymerase Readymix (Kapa Biosystems), 1.25 μl of 5% DMSO, 0.75 μl forward primer, and 0.75 μl reverse primer. PCR cycling was performed by initial denaturation at 95° C for 3 minutes, followed by 35 cycles of 98° C for 20 s, 50° C for 30 s, and 72° C for 30 s, followed by a final extension of 72° C for 7min. PCR efficiency was visualised using gel electrophoresis.

PCR reactions of faecal samples contained 1 to 7 μl of DNA template (0.42 ng/μl–4.2 ng/μl) from the final extraction elutions depending on the DNA concentration of each sample. PCR reactions and cycling conditions were the same as described above. PCRs were run twice to test amplification reproducibility.

The sequencing was performed using Illumina MiSeq platform following the guide for metagenomics sequencing library [34]. Sequencing was carried out at the Australian Genome Research Facility (AGRF) in Brisbane (Australia).

### Bioinformatic analysis

Bioinformatic analysis was performed in Qiime (v.1.8) [35]. Only sequences with Phred quality score higher than 20 were retained during demultiplexing in Qiime. The 8,172,674 quality filtered and trimmed sequences with minimum of 189 and maximum of 211 nucleotides in length are fully annotated and publically available for download at MG-RAST database under project ID 4665244.3. Operational Taxonomic Units (OTUs) were picked using Uclust algorithm [36] at 98% identity. Further filtering of abundance table was used to remove OTUs with less than minimum count fraction of 0.01% and removing samples with less than 10,000 sequences per sample. This strict filtering and quality protocols were performed to avoid possible sequencing data contamination and retain most reliable data.

We assigned OTU taxonomy against an arthropod taxa using the online DNA barcoding workbench Biodiversity of Life Database (BOLD) version 3 [37], which is based on a percentage of similarity between a particular OTU sequence and those sequences stored in the online database. Previous studies have suggested that taxa assignments below 97.4%, using the same arthropod amplicon, are potentially erroneous [38], thus we used a conservative threshold of ≥98% similarity as recommended elsewhere (e.g. [13, 39, 40]). The state of Queensland in eastern Australia, where the study was conducted, has 40,679 published sequences of arthropod species (not including crustaceans) in BOLD, representing 28.75% of the records of this country. Such geographically limited records make confident assignment to species level challenging. However, it is questionable to assign an OTU to a higher taxa when the species level is below the similarity threshold [41]. Hence, we follow a conservative taxa assignation system, by ensuring that each taxonomic match in BOLD was previously recorded in Australia, using a number of resources (e.g. [42, 43]. If no records were found on any assigned taxa, we switched to the following match and re-checked again. We classified as ‘unknown’ an OTU which final taxonomic assignment fell below our set threshold, or any for which a match was not found in the database.

### Statistical analyses

We calculated accumulation curves using the number of OTUs in each faecal sample per bird species, by randomizing the samples 9999 times. We further examined dietary richness using the differences in the number of OTUs between species. Additionally, we checked for differences in the frequency of orders present per bird species, based on the average frequency of each arthropod order per faecal sample.

Throughout this study, Permutational analysis of variance (PERMANOVA) was used. PERMANOVA is a statistical test that uses resemblance-based methods for univariate or multivariate analyses [44]. This routine can deal with unbalanced designs and is recommended for small sample sizes [45]. Particularly, PERMANOVA was used to test for between-species differences in the number of OTUs and the frequency of arthropod orders. Two methods were used to analyse dietary composition, the first one using the number of sequences per OTU and a second one with the presence/absence OTU data. These two methods allowed us to explore any potential biases introduced by sequence abundance per OTU. For each dataset, a similarity matrix based on either Bray-Curtis distances (multiple factors) or Euclidean distances (single factor) was calculated [44]. All PERMANOVA tests were ran using unrestricted permutation of data and 9999 permutations [46]. In those analyses which yielded significant differences, PERMDISP was used to test the homogeneity of dispersion among groups, by calculating the distance to group centroids [47]. Pairwise tests were used for comparisons between groups. Principal coordinates analysis (PCO) [48] based on Bray-Curtis distances, were used to visualize the spatial distribution of the dietary composition of birds in two axes.

Accumulation curves, PERMANOVA, PERMDISP, and PCO, were calculated with PRIMER software (v.6.0) with the PERMANOVA+ add-on (v.1.0.6) (PRIMER-E, Plymouth Marine Laboratory, UK).

## Results

### Overview of the data

We captured 82 individuals from 13 bird species: Lewin's Honeyeater (*Meliphaga lewinii*) (n = 29 individuals), Eastern Yellow Robin (*Eopsaltria australis*) (13), Little Shrike-Thrush (*Colluricincla megarhyncha*) (12), White-throated Treecreeper (*Cormobates leucophaea*) (7), Fairy Gerygone (*Gerygone palpebrosa*) (6), Varied Triller (*Lalage leucomela*) (4), Red-browed Finch (*Neochmia temporalis*) (3), White-throated Honeyeater (*Melithreptus albogularis*) (2), White-browed Scrubwren (*Sericornis frontalis*) (2), Brown Honeyeater (*Lichmera indistincta*) (1), Fan-tailed Cuckoo (*Cacomantis flabelliformis*) (1), Grey Shrike-thrush (*Colluricincla harmonica*) (1), and Silvereye (*Zosterops lateralis*) (1). Only species represented with n≥4 were included in the analysis.

**Fig 1:**
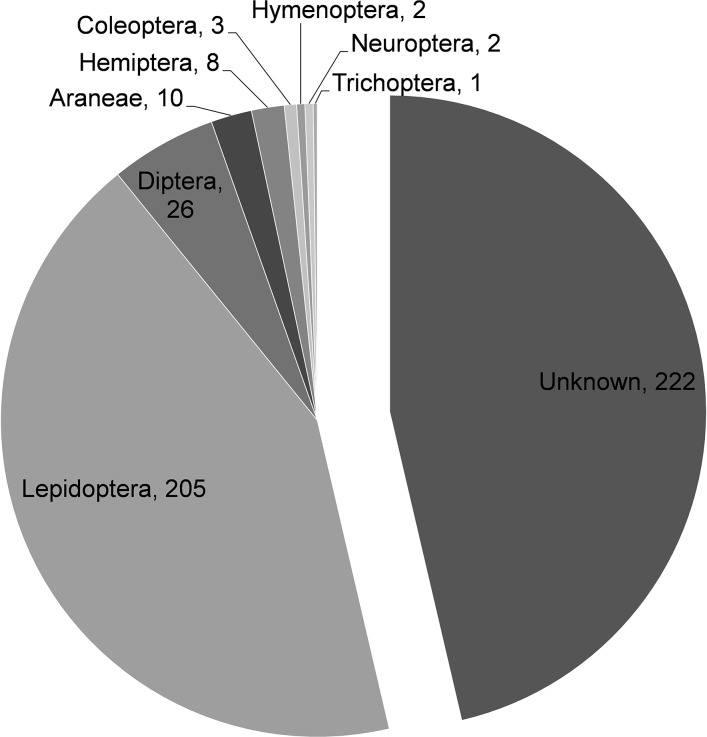
Arthropod orders found on faecal samples (based on ≥ 98% similarity with BOLD sequences). Numbers of Operational Taxonomic Units (OTUs) per order are shown.

From all OTUs, 9 were highly frequent in the dataset (i.e. present in ≥95% of all faecal samples) and represented 31.74% of all the sequences. These 9 OTUs belonged to three lepidopteran families; Oecophoridae (19.91% of sequences), Saturniidae (11.04%), and Bombycidae (0.80%). One hundred and forty six OTUs occurred infrequently (<10% of all faecal samples; 5.51% of all sequences). After all OTUs were blasted against BOLD database based on ≥98% similarity, 257 OTUs (i.e. 53.65% of all OTUs and 61.28% of all sequences) were assigned to an arthropod taxon, while no taxonomic match was found for 222 OTUs (46.35% of all OTUs and 38.72% of all sequences) (Fig 1). The most common orders were Lepidoptera (54.63% of all sequences), Diptera (3.37%) and Araneae (1.09%) (Fig 1).

**Fig 2:**
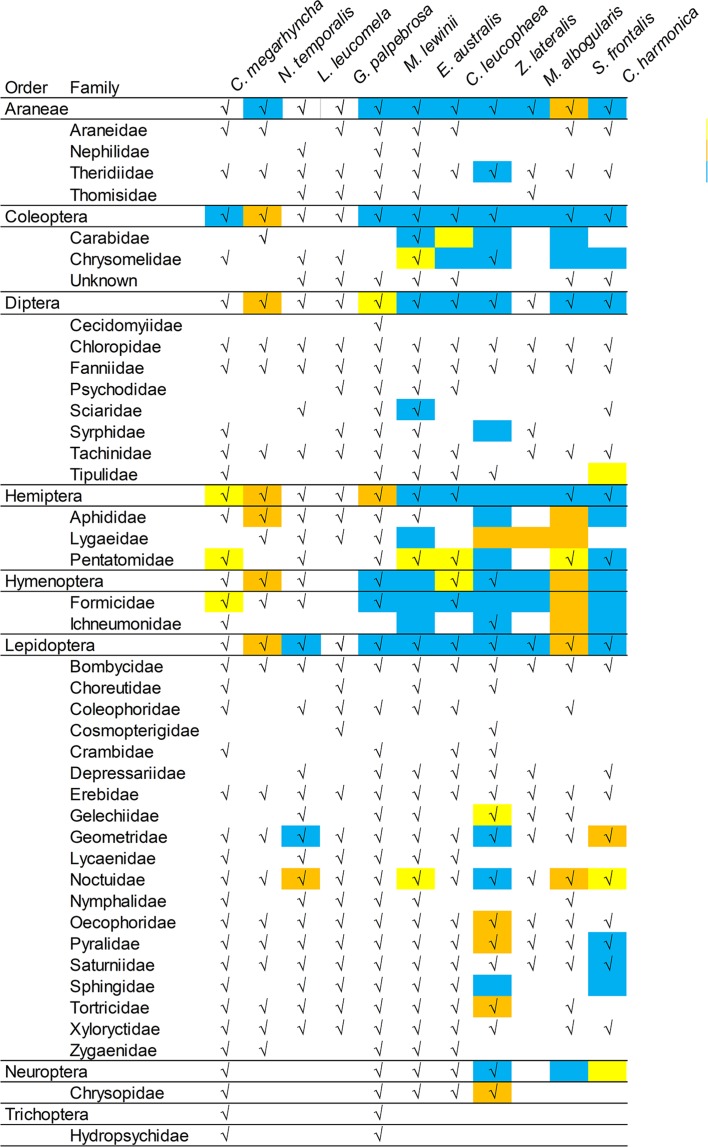
Summary of arthropod consumption by each bird species and comparisons with records found in the Australian literature on avian diet. Note:“√”means that the taxa was recorded in this study. Highlighted in yellow are those records found in The Food of Australian Birds [22, 23], and in orange those found in the Handbook of Australian, New Zealand and Antarctic Birds (HANZAB) [49–51]. Light blue represents those found in both references.

All assigned OTUs were taxonomically divided into 8 orders and 40 families (Fig 2). Lepidopteran had the highest number of families (19) among all orders. Ten families were found in the diet of all bird species: 7 lepidopteran families (i.e. Bombycidae, Erebidae, Geometridae, Noctuidae, Oecophoridae, Pyralidae and Saturniidae), 2 dipteran families (i.e. Chloropidae and Fanniidae) and 1 Araneae family (i.e. Theridiidae). Overall, 21 of the arthropod families were not previously reported in the diets of the studied bird species, according to the leading Australian literature on avian diet [22, 23, 49–51] (Fig 2)

**Fig 3:**
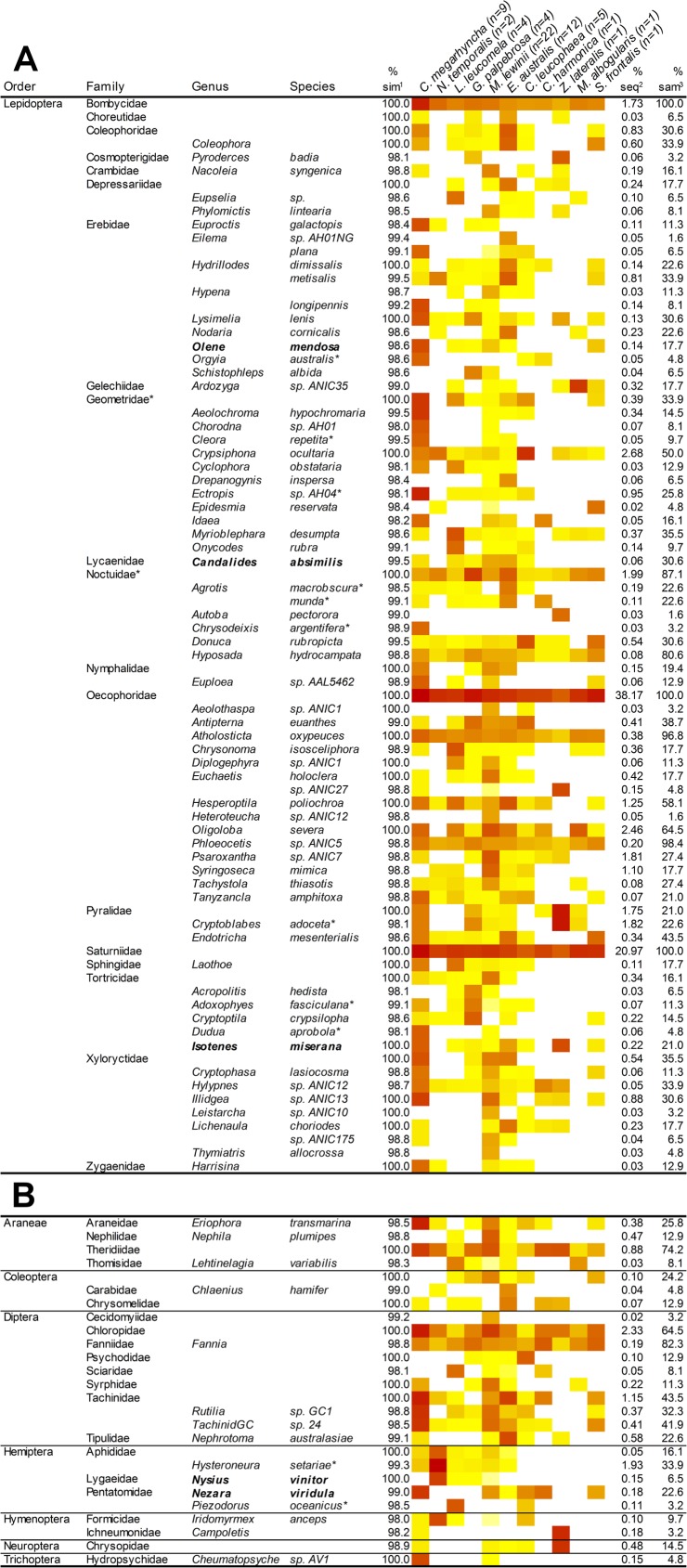
**Abundance of assigned lepidopteran (A) and other than lepidopteran (B) taxa (based on ≥ 98% similarity in BOLD) per bird species. White and dark red colours represent the minimum and maximum number of sequences, respectively. The intensity of the colours across this range represent an increment in the number of sequences.** Note: macadamia pests appear in bold letters and ‘*’ indicates pests of other crops; ‘^1^’ indicates the percentage of similarity on BOLD, ‘^2^’ the percentage of sequences over the total, and ‘^3^’ the percentage of samples containing the taxa over the total.

Seventy out of 90 genera we found (77.78%) belonged to the order Lepidoptera. Three lepidopteran species were present in the diet of all bird species (*Hyposada hydrocampata*, *Atholosticta oxypeuces*, and *Phloeocetis sp*. *ANIC5*) (Fig 3A).

### Insect pests present in the bird’s diet

Among the arthropod taxa found in faecal samples, we found 5 pests of macadamia: *N*. *viridula*, *Nysius vinitor* (Lygaeidae; rutherblen bug), *Olene mendosa* (Erebidae; brown tufted caterpillar), *Candalides absimilis* (Lycaenidae; pencilled blue butterfly), and *Isotenes miserana* (Tortricidae; orange fruitborer) (Fig 3A and 3B). Furthermore, we found 10 arthropod taxa considered pests of other crops: two hemipteran, one from the Aphididae family, *Hysteroneura setariae* (rusty plum aphid) [52], and one from the Pentatomidae family, *Piezodorus oceanicus* (redbanded shield bug) [53], and 8 lepidopteran: One from the Erebidae family, *Orgyia australis* (red tussock moth) [54], 2 from the Geometridae family, *Cleora repetita* (grey looper) and *Ectropis* spp. (loopers) [55, 56], 2 from the Noctuidae family, *Agrotis munda* (brown cutworm) [57] and *Chrysodeixis argentifera* (tobacco looper) [58], 1 from the Pyralidae family, *Cryptoblabes adoceta* (sorghum head caterpillar) [59], and 2 from the Tortricidae family, *Adoxophyes fasciculana* (bell moth) [60] and *Dudua aprobola* (tortrix moth) [61] (Fig 3A and 3B).

### Birds’ dietary richness

**Fig 4:**
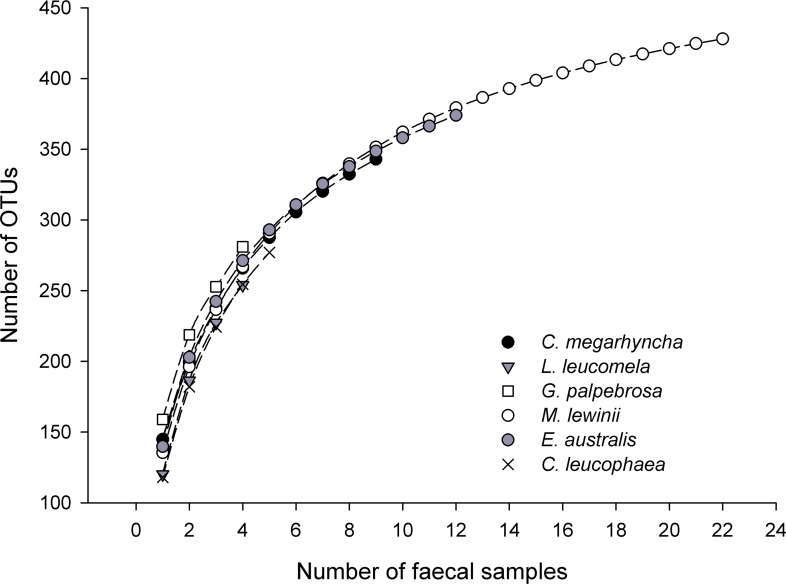
OTUs accumulation curves of each bird species.

The OTUs accumulation curves were similar between bird species, showing maximum increases in the cumulative number of OTUs during the first three faecal samples, when all bird species were in a range of approximately 220 to 250 OTUs (i.e. equivalent to approximately 50% over the total number of OTUs) (Fig 4).

**Fig 5:**
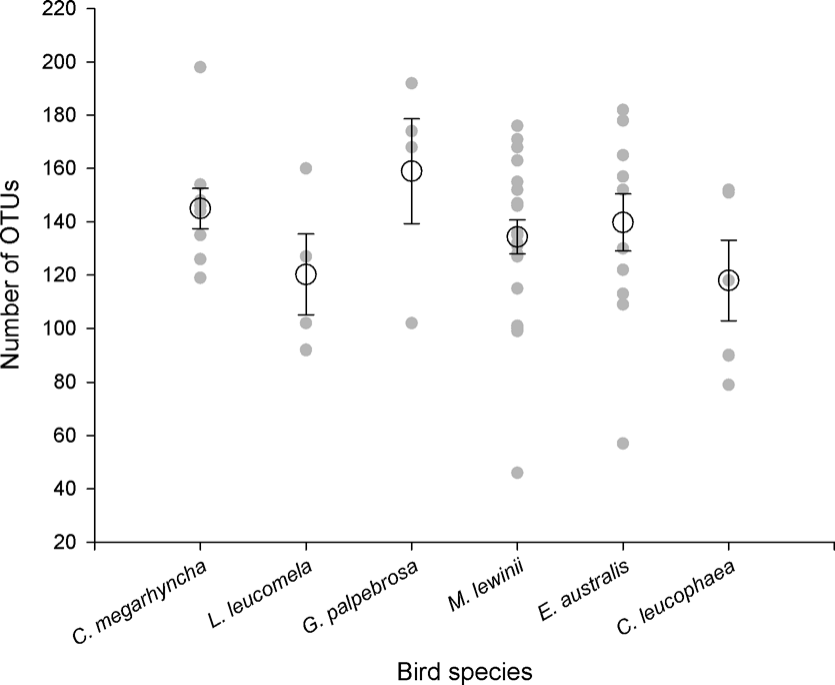
Number of OTUs found in the faecal samples of each bird species. Grey dots represent the number of OTUs in a faecal sample, and larger circles represent the average value (± SE) of individual bird species.

The number of OTUs ranged from a minimum of 118 in *C*. *leucophaea* to a maximum of 159 in *G*. *palpebrosa* (Fig 5) (although *C*. *harmonica* and *S*. *frontalis*, both not considered in the analyses due to low faecal sample size, obtained 173 and 207 respectively). Results showed non-significant differences in the number of OTUs between species (Pseudo-F = 1.135, P = 0.348).

### Comparison of the dietary compositions

**Fig 6:**
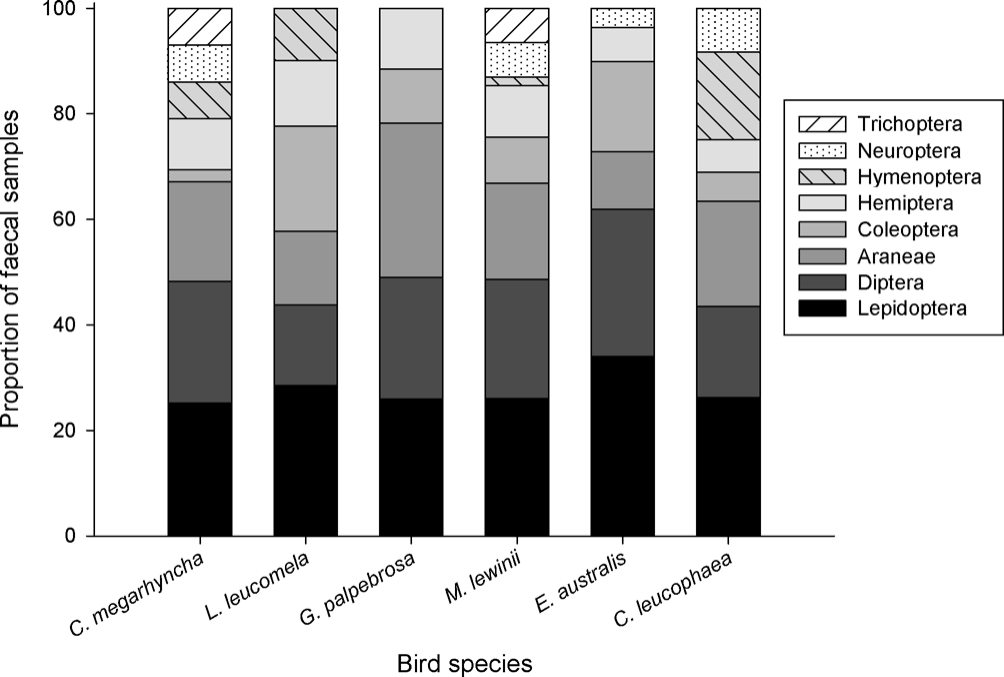
Frequency of arthropod orders in the diet of bird species, based on percentages of presence in faecal samples.

The proportion of OTUs taxonomically assigned to Lepidoptera differed significantly between bird species (Pseudo-F = 2.315, P = 0.035). However, PERMDISP results were also significant (Pseudo-F = 7.345, P = 0.001), showing dispersion in the data. No significant differences were found in the frequency of any other order (Fig 6). Five arthropod orders (i.e. Lepidoptera, Diptera, Araneae, Coleoptera and Hemiptera) were present in all bird species. Both *M*. *lewinii* and *C*. *megarhyncha* were the only two species with diets containing all 8 recorded arthropod orders, including Trichoptera which was only present in those two bird species. The *G*. *palpebrosa* diet contained 5 orders, the lowest value among all species (Fig 6).

**Fig 7:**
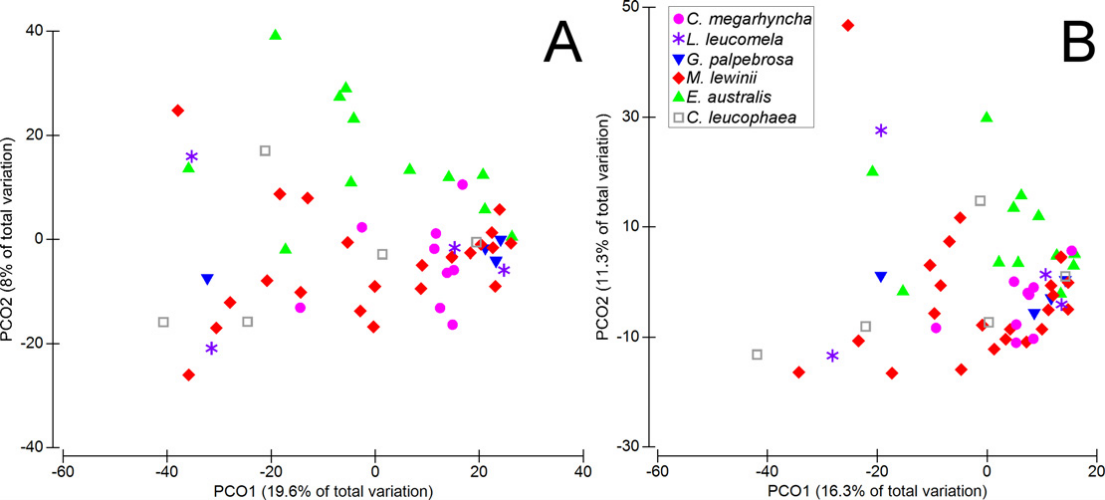
Principal coordinates analysis (PCO) based on Bray Curtis similarity showing unconstrained ordination of log-transformed data (A) and presence-absence data (B).

The diet composition, based on the number of sequences per OTU, differed significantly between bird species (Pseudo-F = 1.765 P = 0.001). Pairwise tests showed significant differences between the diet composition of *E*. *australis* and those of *G*. *palpebrosa* (t = 1.379 P = 0.037), *M*. *lewinii* (t = 1.655, P = 0.001), *C*. *megarhyncha* (t = 1.539, P = 0.001), and *C*. *leucophaea* (t = 1.350, P = 0.022), and also between the diet composition of *C*. *megarhyncha* and those of *G*. *palpebrosa* (t = 1.417, P = 0.015), *L*. *leucomela* (t = 1.344, P = 0.025), and *C*. *leucophaea* (t = 1.429, P = 0.010). When presence/absence data were used, PERMANOVA results were similar to those obtained when using the number of sequences per OTU, showing significant differences in dietary composition between bird species (Pseudo-F = 1.622 P = 0.003). Only one pairwise test comparison (*E*. *australis* and *G*. *palpebrosa*, t = 1.273, P = 0.073) produced a non-significant result, in contrast with the other method. Significant differences were found between the dietary composition of *E*. *australis* and those of *M*. *lewinii* (t = 1.6112, P = 0.001), *C*. *megarhyncha* (t = 1.498, P = 0.001), and *C*. *leucophaea* (t = 1.311, P = 0.038), and also between that of *C*. *megarhyncha* and those of *G*. *palpebrosa* (t = 1.399, P = 0.018), *L*. *leucomela* (t = 1.291, P = 0.049), and *C*. *leucophaea* (t = 1.372, P = 0.029). PERMDISP results confirmed that differences in dietary composition between bird species obtained with PERMANOVA were due to multivariate location, and not to multivariate dispersion (log-transformed data: F = 1.632, P = 0.486; presence-absence data: F = 1.590, P = 0.469). Between-species differences showed in PERMANOVA analyses were visualized using PCO ordination plots, where the first two axes explained a total variation of 27.6% on both log-transformed and presence-absence datasets (Fig 7).

## Discussion

This is the first study using NGS and bioinformatics analyses to explore the diet of birds and associated ecosystem services by sequencing the arthropod DNA present in their faeces. Using the MiSeq platform of Illumina, we successfully sequenced DNA in 62 faecal samples from 11 bird species foraging in macadamia agro-ecosystems. Our results revealed that avian communities deliver pest-reduction services in this system. This study showed differences in dietary composition, but most of the bird species had wide insectivorous diets and shared a large proportion of prey items, indicating a low resource partitioning in these systems.

### Insect pests found in bird’s diets

We report the presence of 5 macadamia-specific insect pests. Four of these are considered ‘minor’ (*N*. *vinitor*, *O*. *mendosa*, *C*. *absimilis*, and *I*. *miserana*), and 1 ‘major’ *N*. *viridula*. The later species, commonly known as the green vegetable bug, is a threat to macadamia production in Australia [62] and is also considered to be one of the most damaging pests throughout the studied area. It is also considered a major macadamia pest in other countries (e.g. [63, 64]). In the present study, *N*. *viridula* was present in 23% of bird faecal samples. Specifically, it was present in 32% and 25% of all faecal samples from two of the most commonly trapped birds, *M*. *lewinii* and *E*. *australis*, respectively. In general, its presence was recorded in 6 out of 11 bird species. *Nezara viridula* seasonally alternates between crop and non-crop plant species [65], and previous studies in the area have recorded it in both macadamia orchards and adjacent woodland (Crisol-Martínez, unpublished work), thus birds in woodland-macadamia interfaces can potentially prey on them in both habitats. Our results show that most birds consumed this species opportunistically, although its relatively higher presence in faecal samples from both *M*. *lewinii* and *E*. *australis* makes these two species the most important ones from a pest-management point of view. Unfortunately, even though *A*. *lutescens lutescens* presence was recorded during insect pest surveys, its DNA could not be amplified with the arthropod-specific primers used in this study, thus species-specific primers need to be developed in future studies in order to understand whether this pest is consumed by avian communities.

Although at present it is not possible to quantify the number of individuals of any eaten insect pest using NGS [66], our results have confirmed the delivery of pest-reduction services in macadamia orchards by avian communities. A number of arthropod predators such as arachnids were found in the diet of most bird species, yet it is unclear whether this intraguild predation (i.e. predation of predators) could weaken any potential trophic cascades caused by avian communities in macadamia orchards. A review by Mantyla *et al*. [5] showed numerous examples in which avian communities composed by generalist predators indirectly helped plants through trophic cascades in several ecosystem types, including agro-ecosystems. Furthermore, a number of authors (e.g. [67, 68]) suggested that avian trophic cascades are stronger in simple ecosystems such as agricultural environments. Further research is necessary to clarify the scale of the pest-reduction contribution of avian communities in macadamia orchards.

Additionally, our results indicate the presence of 10 insect pests from other crops in the diet of the studied birds. Some of these pests can damage economically important crops present in the study area such as sugarcane (*H*. *setariae*) [69], summer pulses (*P*. *oceanicus*, *O*. *australis*, *C*. *argentifera*, *C*. *adoceta*) [53, 58, 59], lychee and mango (*D*. *aprobola*) [61], avocado (*Ectropis* spp., *C*. *repetita*) [55, 56], and strawberries (*Agrotis* spp.) [70]. These results highlight the importance of conserving avian communities in agricultural landscapes in the study area. Further research in macadamia agro-ecosystems is necessary to elucidate whether avian communities can significantly decrease the pest pressure in macadamia and other crops, to understand whether these pest-reduction services indirectly promote increases in yield, and to calculate an economic estimation of these ecosystem services.

### Analysis and comparison of bird species’ diets

Species accumulation curves indicated that, most likely due to low sample sizes, we were not able to fully describe the entire insectivorous diet of any of the bird species. We found no significant differences in the number of OTUs between bird species. The frequency of individual arthropod orders found in faecal samples did not show differences between bird species, suggesting that the majority of avian species foraging in riparian woodland-macadamia interfaces shared food resources, by feeding primarily on lepidopteran, dipteran and arachnids. Several records of birds in riparian habitats show that the tree canopy is the most used habitat stratum [71], supporting these general dietary similarities between sympatric species.

Permutational analyses allowed us an in-depth comparison of the dataset, showing significant differences in dietary composition between specific bird species. Overall, there were not differences in the results found when using the number DNA sequences per OTU or the presence/absence of OTUs, indicating that DNA abundance per OTU did not bias the results. Particularly, the dietary composition of *E*. *australis* differed from most species, as visually shown by the cluster formed by its samples in the PCO plots. These differences are likely due to the foraging behaviour of *E*. *australis*, considered mostly a ground-forager, as opposed to the other species, which are mostly arboreal [49–51]. Additionally, *C*. *megarhyncha* showed a different dietary composition compared to those of *L*. *leucomela*, *E*. *australis*, *G*. *palpebrosa* and *C*. *leucophaea*. Previous studies in northern Australia found that *C*. *megarhyncha* showed higher foraging habitat diversity than a number of other bird species [72, 73]. Another Australian study found that *C*. *megarhyncha* showed higher foraging plasticity across vegetation types than other bird species, including *E*. *australis*, and *C*. *leucophaea*, whose numbers were biased towards particular habitats [74]. These studies suggest that *C*. *megarhyncha* has a high foraging plasticity, which is in accordance with the differences in diet composition with other species found in this study. Although *L*. *leucomela* was, together with *C*. *megarhyncha*, characteristic of the same riparian zone class in northern Australia [26], the diet of the former species includes insects, seeds and nectar [75], as opposed to the insectivorous habits of *C*. *megarhyncha* [72]. Also, *C*. *leucophaea* dietary composition differed from those of *C*. *megarhyncha* and *E*. *australis*. These three species are insectivorous, but a number of studies have reported the presence of a majority of ants in the diet of *C*. *leucophaea* (e.g. [76, 77]). Our results agreed, showing higher frequency of occurrence of Formicidae in *C*. *leucophaea* than in the other two species. Furthermore, based on the average number of OTUs, the dietary richness was the lowest in *C*. *leucophaea*. The PCO plots showed that samples from *M*. *lewinii* were spatially spread, overlapping with most avian species, suggesting a generalist behaviour. Specialists such as the *C*. *leucophaea*, a ‘bark-feeder’ species [78], tend to have narrower niche breadth than generalists [79], such as *M*. *lewinii*, considered a mixed-feeder species [80].

### Opportunities and limitations

While NGS offers a powerful tool to analyse the diet of wildlife, it also presents a number of limitations, such as the impossibility of determining whether any arthropod’s DNA detected is a result of primary or secondary predation [81]. It is also currently impossible to identify the life stage of the preys [82], which could elucidate the pest-reduction services provided by birds upon predation on the most damaging pest developmental stages. The other potential source of bias is the differences in digestion rates between soft- and hard-bodied arthropods. However, it is well established that birds have only a few hours of digesta passage rate due to their unique intestinal morphology adapted to accommodate high energy needs for flight [83]. With feed remaining in the gut for only few hours [84], the freshly collected faecal samples will contain their latest meals at different stage at digestion, yet most likely with enough DNA to make soft-bodied arthropods detectable with PCR amplification. Each arthropod cell will contain the marker gene and the size of the arthropod will influence the number of cells and consequently the number of sequences detected. Thus we must have in mind that the present sequencing-based technique, as any other marker sequencing method, cannot quantify the amount of any specific taxa [66], yet it can only provide an estimation of the frequency of occurrence, allowing comparisons of relative proportions of consumed taxa between treatments reliably. We cannot therefore definitely distinguish the bird preferences for specific arthropod species over others based on higher sequence counts. To increase the quality of future dietary studies, it is recommended to combine NGS and traditional methods, as other authors have suggested [85].

An additional limitation is the number of arthropod sequences characterised for the COI region in BOLD or any other online database, which constrains the reconstruction of the diet of the studied birds. Nevertheless, these databases are growing at an incredible speed [10] and NGS-based techniques are revolutionising our understanding of life on the planet. With the falling cost of sequencing and continual growth in the number of manuscripts published, NGS is making a remarkable impact on all areas of life science, especially ecology.

## Conclusions

The data from the study clearly demonstrate the applicability of NGS analyses to explore the diet of birds and infer ecosystem functions. The diet composition comparisons based on NGS data were consistent with known foraging behaviours of the captured avian species. Additionally, the species-specific diet description at order and family levels using NGS is consistent with previous records based on traditional methods [23, 24, 49–51], thus reinforcing our results. Moreover, this study expands these records with a large number of arthropod families, genera and species previously unreported in the literature, particularly lepidopterans and dipterans, and especially in the case of those birds whose diet is understudied, such as *G*. *palpebrosa*, *L*. *leucomela*, and *C*. *megarhyncha*.

The dietary analysis of the studied bird species is in accordance with the research previously published about their foraging behaviour. Because of their rich vegetation structure, remnant riparian habitats provide a wide range of foraging strata that maximise the bird guilds inhabiting them [86]. Guild-rich avian communities consume a diverse array of arthropods, which may translate into improved pest-reduction services in agricultural land, ultimately leading to bird conservation in those areas when the diverse vegetation structure is maintained.

Overall, our study showed that NGS using the MiSeq Illumina platform and subsequent bioinformatics analyses offer a solid, relatively fast and recently increasingly affordable way to explore the diet of mixed-guild avian communities, delivering a wide scope and detailed resolution on the prey taxonomic level, which offers an opportunity to better understand their diet. Our results highlight the benefits that conserving birds foraging in orchards can bring to the macadamia industry and stress the need to protect the riparian habitats in this region to maintain avian richness.
